# Crystal structures and the Hirshfeld surface analysis of *(E)*-4-nitro-*N*′-(*o*-chloro, *o*- and *p*-methyl­benzyl­idene)benzene­sulfono­hydrazides

**DOI:** 10.1107/S2056989018015207

**Published:** 2018-11-06

**Authors:** Akshatha R. Salian, Sabine Foro, B. Thimme Gowda

**Affiliations:** a Department of Chemistry, Mangalore University, Mangalagangotri-574 199, India; b Institute of Materials Science, Darmstadt University of Technology, Alarich-Weiss-Str. 2, D-64287, Darmstadt, Germany; c Karnataka State Rural Development and Panchayat Raj University, Raitha Bhavan, Gadag-582101, India

**Keywords:** crystal structure, *N*-(aryl­idene)-aryl­sulfono­hydrazides, hydrogen bonding, ring motif, Hirshfeld surface analysis, fingerprint plots

## Abstract

The crystal structures of (*E*)-*N*′-(2-chloro­benzyl­idene)-4-nitro­benzene­sulfono­hydrazide, C_13_H_10_ClN_3_O_4_S (I), (*E*)-*N*′-(2-methyl­benzyl­idene)-4-nitro­benzene­sulfono­hydrazide, C_14_H_13_N_3_O_4_S (II), and (*E*)-*N*′-(4-methyl­benzyl­idene)-4-nitro­benzene­sulfono­hydrazide monohydrate, C_14_H_13_N_3_O_4_S·H_2_O (III), have been synthesized, characterized and their crystal structures determined to study the effects of the nature and sites of substitutions on the structural parameters and the hydrogen-bonding inter­actions. All three compounds crystallize in the monoclinic crystal system, with space group *P*2_1_ for (I) and *P*2_1_/*c* for (II) and (III). Compound (III) crystallizes as a monohydrate. All three compounds adopt an *E* configuration around the C=N bond. The mol­ecules are bent at the S atom with C—S—N—N torsion angles of −59.0 (3), 58.0 (2) and −70.2 (1)° in (I), (II) and (III), respectively. The sulfono­hydrazide parts are also non-linear, as is evident from the S—N—N—C torsional angles of 159.3 (3), −164.2 (1) and 152.3 (1)° in (I), (II) and (III), respectively, while the hydrazide parts are almost planar with the N—N=C—C torsion angles being −179.1 (3)° in (I), 176.7 (2)° in (II) and 175.0 (2)° in (III). The 4-nitro-substituted phenyl­sulfonyl and 2/4-substituted benzyl­idene rings are inclined to each other by 81.1 (1)° in (I), 81.4 (1)° in (II) and 74.4 (1)° in (III). The compounds show differences in hydrogen-bonding inter­actions. In the crystal of (I), mol­ecules are linked *via* N—H⋯O hydrogen bonds, forming *C*(4) chains along the *a-*axis direction that are inter­connected by weak C—H⋯O hydrogen bonds, generating layers parallel to the *ac* plane. In the crystal of (II), the amino H atom shows bifurcated N—H⋯O(O) hydrogen bonding with both O atoms of the nitro group generating *C*(9) chains along the *b*-axis direction. The chains are linked by weak C—H⋯O hydrogen bonds, forming a three-dimensional framework. In the crystal of (III), mol­ecules are linked by O*w*—H⋯O, N—H⋯O*w* and C—H⋯O hydrogen bonds, forming layers lying parallel to the *bc* plane. The fingerprint plots generated for the three compounds show that for (I) and (II) the O⋯H/H⋯O contacts make the largest contributions, while for the *para*-substituted compound (III), H⋯H contacts are the major contributors to the Hirshfeld surfaces.

## Abstract 2

The crystal structures of three *N′*-(aryl­idene)4-nitro­benzene­sulfono­hydrazides, namely, (*E*)-4-nitro-*N*′-(2-chloro­benzyl­idene)benzene­sulfono­hydrazide (I), (*E*)-4-nitro-*N*′-(2-methyl­benzyl­idene) benzene­sulfono­hydrazide (II) and (*E*)-4-nitro-*N*′-(4-methyl­benzyl­idene)benzene­sulfono­hydrazide (III), have been studied to investigate the effect of the nature and site of substitutions on the structural parameters and the supra­molecular features in these compounds. Hirshfeld surface analysis was also carried out to examine the contributions of the various atom–atom inter­actions in the crystal packing of the three compounds.

## Chemical context   

Sulfonyl hydrazides have been used extensively to synthesize new Schiff bases owing to the presence of two chemically and biologically important sulfonyl and hydrazine moieties (Murtaza *et al.*, 2016 ▸). Reactions of hydrazines with other functional groups produce compounds with unique physical and chemical characteristics (Xavier *et al.*, 2012 ▸). Hydrazones derived from the condensation reactions of hydrazides with aldehydes show excellent biological properties (Küçükgüzel *et al.*, 2006 ▸). As a result of the ease of the electron-transport mechanism through the *π*-conjugated framework, the azomethine-bridged benzene derivatives exhibit excellent optical non-linearities (Manivannan & Dhanuskodi, 2004 ▸). Organic polymers containing the azomethine group are known to have good mechanical strength (Morgan *et al.*, 1987 ▸) and high thermal stability (Catanescu *et al.*, 2001 ▸). As part of our continuing studies to explore the effect of the nature and site of substituents on the crystal structures of sulfonyl hydrazide derivatives (Salian *et al.*, 2018 ▸), we report herein the synthesis, crystal structures and Hirshfeld surface analyses of the title compounds, (*E*)-4-nitro-*N*′-(2-chloro­benzyl­idene)benzene­sulfono­hydrazide (I), (*E*)-4-nitro-*N*′-(2-methyl­benzyl­­idene)benzene­sulfono­hydrazide (II) and (*E*)-4-nitro-*N*′-(4-methyl­benzyl­idene)benzene­sulfono­hydrazide monohydrate (III).
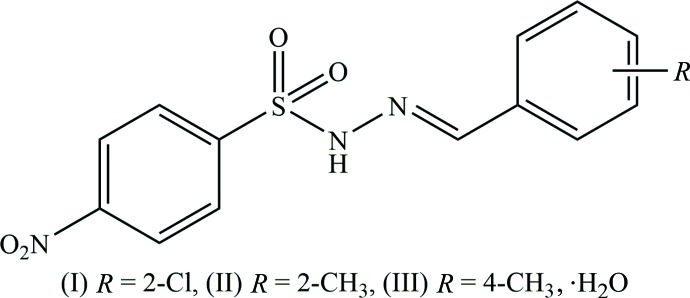


## Structural commentary   

**Figure 1:**
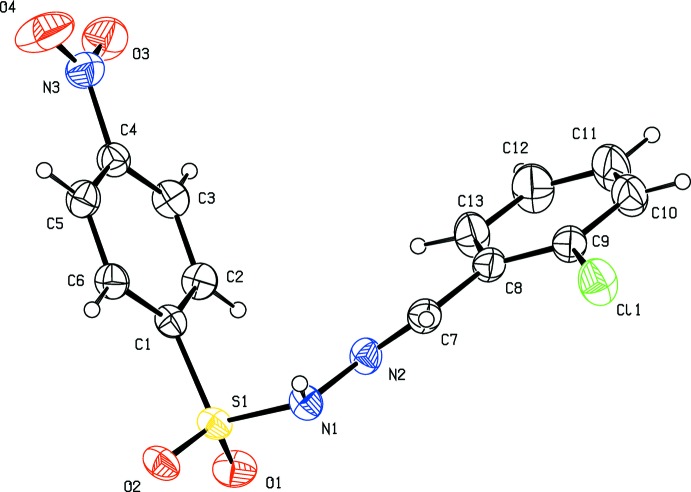
Mol­ecular structure of compound (I), showing the atom labelling and displacement ellipsoids drawn at the 30% probability level.

The title compounds crystallize in the monoclinic crystal system, in space group *P*2_1_ for (I) and *P*2_1_/c for (II) and (III). The mol­ecular structures of the three compounds are shown in Figs. 1 ▸, 2 ▸ and 3 ▸. Compound (III) crystallizes as a monohydrate. In all three compounds the configuration about the C=N bond is *E*, with C7=N2 bond lengths of 1.269 (5), 1.275 (2) and 1.263 (3) Å in (I), (II) and (III), respectively. The respective N1—N2 bond lengths of 1.404 (4), 1.400 (2) and 1.398 (2) Å are consistent with the azine bond lengths of 1.40 Å in similar structures (Salian *et al.*, 2018 ▸), indicating the delocalization of *π*-electron density over the hydrazone portion of the mol­ecules. The conformation of the N—H and C—H bonds in (I) and (II), with respect to the *ortho*-substit­uents, are *syn* to each other (Figs. 1 ▸ and 2 ▸). In the central parts of each mol­ecule the S1—N1—N2=C7 torsion angles deviate from linearity with values of 159.3 (3)° in (I), −164.2 (1)° in (II) and 152.3 (1)° in (III), while the hydrazide parts are almost planar with the N1—N2=C7—C8 torsion angles being −179.1 (3), 176.7 (2) and 175.0 (2)° in (I), (II) and (III), respectively. The dihedral angles between the 4-nitro­benzene ring (C1–C6) and benzene ring (C8–C13) are 81.1 (1), 81.4 (1) and 74.4 (1)°, respectively. The plane of the nitro group (N3/O3/O4) is inclined to the 4-nitro­benzene ring (C1–C6) by 9.3 (5) ° in (I) and 9.1 (3) ° in (II), but is significantly out of plane in (III) with a dihedral angle of 16.1 (2)°.

## Supra­molecular features   

**Figure 4:**
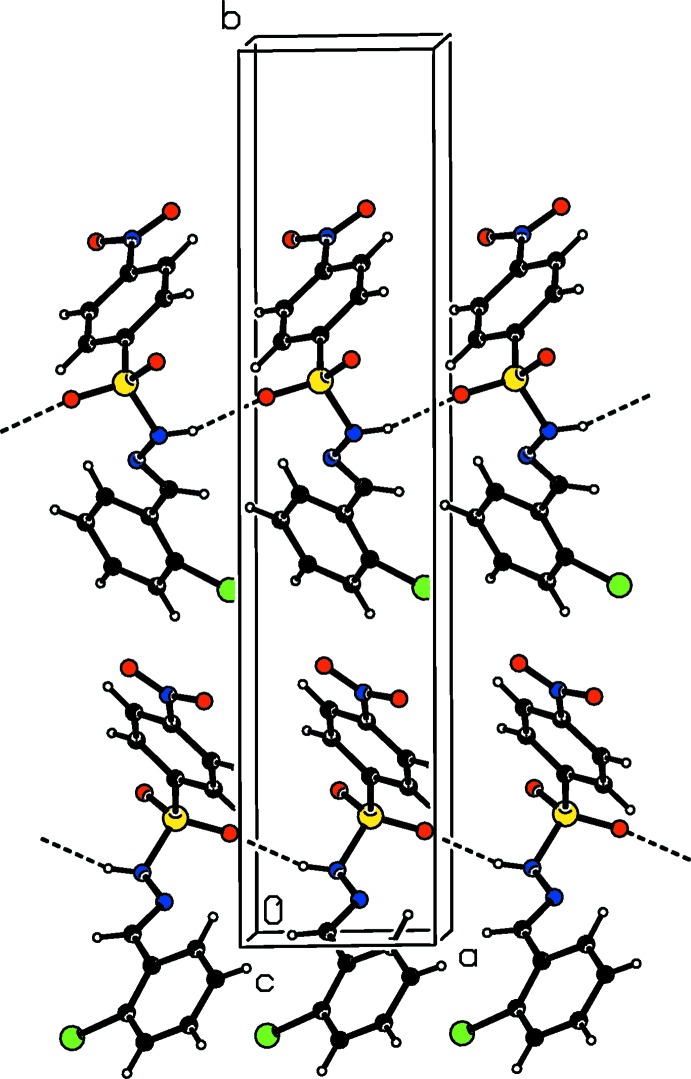
A partial view along the *c* axis of the crystal packing of compound (I), with hydrogen bonds shown as dashed lines.

**Figure 5:**
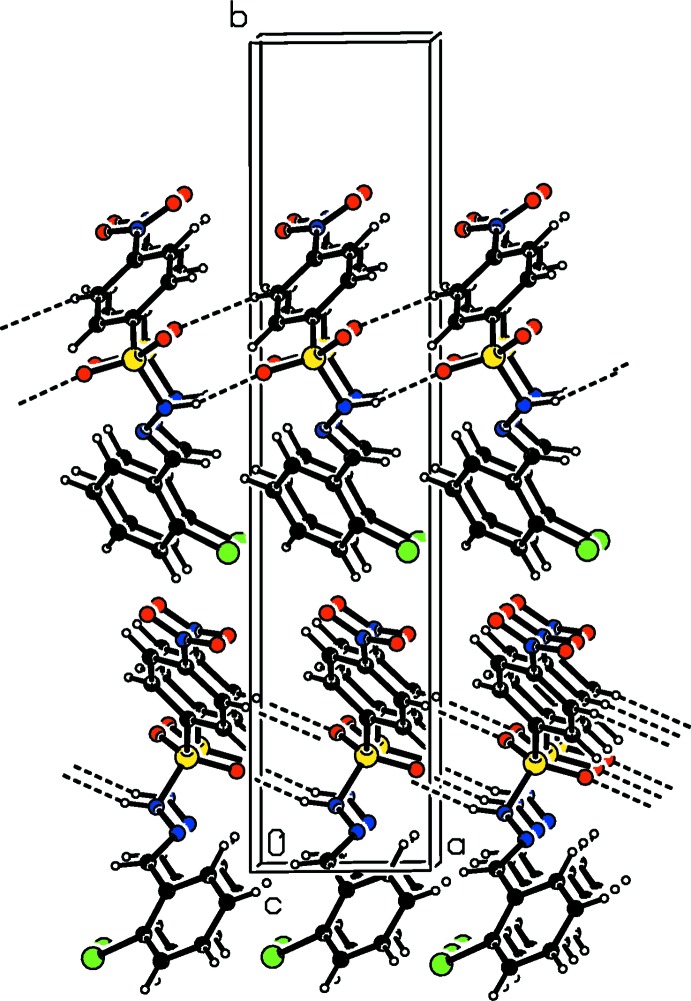
The crystal packing of compound (I), viewed along the *c* axis, with hydrogen bonds shown as dashed lines.

**Figure 6:**
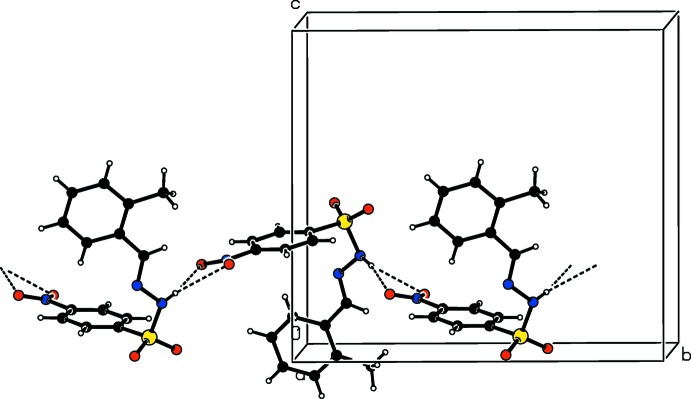
A partial view along the *a* axis of the crystal packing of compound (II), with hydrogen bonds shown as dashed lines.

**Figure 7:**
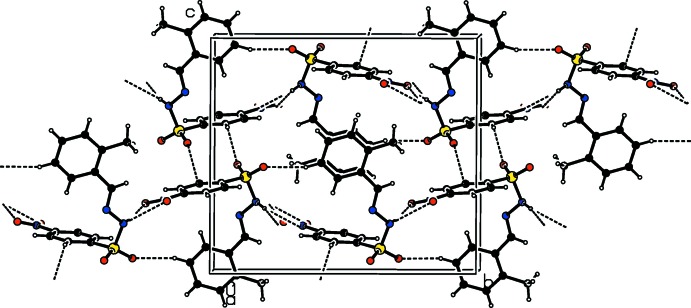
The crystal packing of compound (II), viewed along the *a* axis,with hydrogen bonds shown as dashed lines.

**Figure 8:**
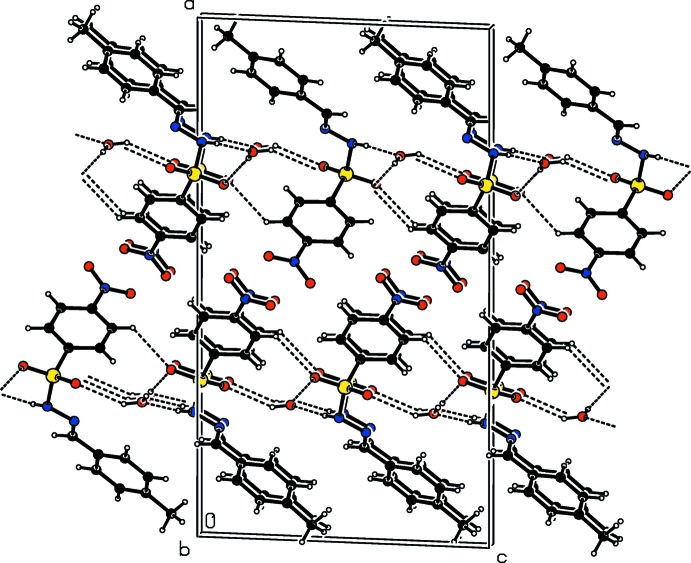
The crystal packing of compound (III), viewed along the *b* axis, with hydrogen bonds shown as dashed lines.

In the crystals of the title compounds there are significant difference in the hydrogen-bonding inter­actions. In the crystal of the *ortho*-chloro-substituted compound (I), mol­ecules are linked *via* N—H⋯O hydrogen bonds, forming *C*4 chains along the *a*-axis direction (Table 1 ▸ and Fig. 4 ▸). These chains are inter­connected by weak C—H⋯O hydrogen bonds, generating layers parallel to the *ab* plane (Table 1 ▸ and Fig. 5 ▸). In the crystal of the *ortho*-methyl-substituted compound (II), the amino H atom shows bifurcated N—H⋯O(O) hydrogen bonding with both the O atoms of the nitro group, generating chains with a *C*(9) motif that propagate along the *b*-axis direction (Table 2 ▸ and Fig. 6 ▸). These chains are linked by C—H⋯O hydrogen bonds, resulting in the formation of a three-dimensional framework (Table 2 ▸ and Fig. 7 ▸). Finally, in the crystal of the *para*-methyl-substituted compound (III), the presence of the water mol­ecule of crystallization has an important effect on the crystal packing. The mol­ecules of compound (III) are bridged by the water mol­ecule, *via* O*w*—H⋯O and N—H⋯O*w* hydrogen bonds, forming layers lying parallel to the *bc* plane that are reinforced by C—H⋯O hydrogen bonds (Table 3 ▸ and Fig. 8 ▸).

## Hirshfeld surface analysis   

**Figure 9:**
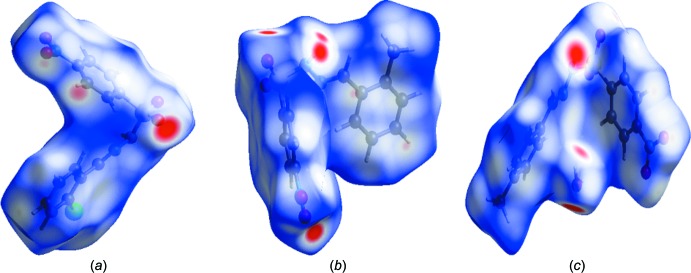
View of the Hirshfeld surface mapped over *d*_norm_ for (*a*) (I), (*b*) (II) and (*c*) (III).

**Figure 10:**
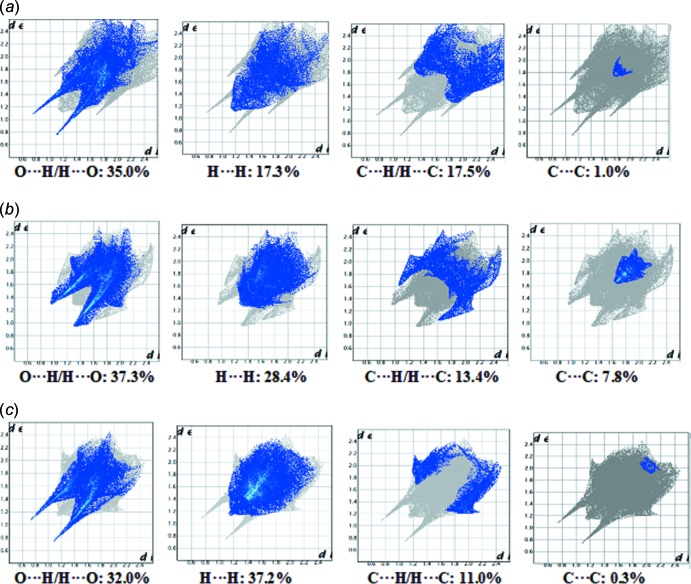
Two-dimensional fingerprint plots showing the contributions of the different types of inter­actions in (*a*) (I), (*b*) (II) and (*c*) (III).

The type of inter­molecular contacts and their qu­anti­tative contribution to the crystal packing in all the three compounds were studied by Hirshfeld surfaces and two-dimensional fingerprint plots, which were generated using *CrystalExplorer3.1* (Wolff *et al.*, 2012 ▸). The Hirshfeld surfaces mapped over *d*_norm_ are in the scale of −0.56–1.43 a.u. The bright-red spots on the Hirshfeld surfaces mapped over *d*_norm_ indicate the strong N—H⋯O inter­actions present in the crystal structure (McKinnon *et al.*, 2004 ▸; Spackman & Jayatilaka, 2009 ▸); these correspond to N1—H1*N*⋯O1^i^ in (I) (Fig. 9 ▸ *a*; Table 1 ▸), N1—H1*N*⋯O3^i^ and N1—H1*N*⋯O4^i^ in (II) (Fig. 9 ▸ *b*; Table 2 ▸) and N1—H1*N*⋯O5^i^, O5—H51⋯O2^ii^ and O5—H52⋯O1^iii^ in (III) (Fig. 9 ▸ *c*; Table 3 ▸). The fingerprint plots corresponding to the various contacts contributing more than 10% (along with the C⋯C contacts) to the Hirshfeld surfaces are shown in Fig. 10 ▸. Table 4 ▸ lists all the contacts present in the crystal structures of the three compounds, and their respective percentage contributions to the Hirshfeld surfaces. The O⋯H/H⋯O contacts correspond to the N—H⋯O/O—H⋯O inter­actions at *d*_e_ + *d*_i_ ∼2.2 Å in (I) and (III) and at 2.6 Å in (II), which is very close to the hydrogen-bonding distances observed in these compounds [Tables 1 ▸, 2 ▸ and 3 ▸ for (I), (II) and (III), respectively]. These inter­actions are the major contributor in (I) and (II) [35.0% in (I) and 37.3% in (II)] followed by H⋯H contacts [17.5% in (I) and 28.4% in (II)]. In (III), H⋯H inter­actions make the largest contribution to the Hirshfeld surface (37.2%), followed by O⋯H/H⋯O contacts (32.0%). The H⋯H inter­actions appear as a short single peak at *d*_e_ + *d*_i_ ∼2.2 Å in the fingerprint plot of (III) (see Fig. 10 ▸ *c*). The distinct pair of wings corresponds to C⋯H/H⋯C contacts, which are the third largest contributor to the Hirshfeld surfaces in all three compounds [17.3% in (I), 13.4% in (II) and 11.0% in (III)]. A significant difference is in the percentage contribution from C⋯C contacts found for the three compounds. They are characterized by two overlapping broad peaks in the fingerprint plot for (II) (see Fig. 10 ▸ *b*), accounting for 7.8% of the Hirshfeld surface with *d*_e_ + *d*_i_ ∼3.4 Å, whereas for (I) and (III) these inter­actions make negligible contributions of 1.0 and 0.3%, respectively. The O⋯C/C⋯O contacts contribute 4.3% in (I), 1.8% in (II) and 9.4% in (III). N⋯H/H⋯N contacts contribute 4.3, 7.3 and 5.0% in (I), (II) and (III), respectively. In (I), the Cl⋯H/H⋯Cl, Cl⋯C/C⋯Cl, Cl⋯O/O⋯Cl and Cl⋯N/N⋯Cl contacts contribute 6.1, 4.7, 3.1 and 1.4%, respectively, to the Hirshfeld surfaces. The percentage contributions for the various inter­actions in the title compounds are compared in Table 4 ▸.

## Database survey   

Structures similar to the title compounds that have been reported in the literature include *N*′-[(*E*)-4-methyl­benzyl­idene]-4-methyl­benzene­sulfono­hydrazide (Tabatabaee *et al.*, 2007 ▸), (*E*)-*N*′-(4-chloro­benzyl­idene)-*p*-toluene­sulfono­hydrazide 0.15-hydrate (Kia *et al.*, 2009*a* ▸), (*E*)-*N*′-(4-chloro­benzyl­idene)-4-methyl­benzene­sulfono­hydrazide (Balaji *et al.*, 2014 ▸), (*E*)-*N*′-(4-bromo­benzyl­idene)-*p*-toluene­sulfono­hydrazide (Kia *et al.*, 2009*b* ▸), (*E*)-*N*′-(4-nitro­benzyl­idene)-benzene­sulfono­hydrazide (Hussain *et al.*, 2017*a* ▸), *E*)-4-methyl-*N′*-(4-nitro­benzyl­idene)benzene­sulfono­hydrazide (Hussain *et al.*, 2017*b* ▸). (*E*)-*N*′-(2-methyl­benzyl­idene)-4-chloro­benzene­sulfono­hydrazide and (*E*)-*N*′-(4-methyl­benzyl­idene)-4-chloro­benzene­sulfono­hydrazide (Salian *et al.*, 2018 ▸). In all the structures, inter­molecular N—H⋯O hydrogen bonds link neighbouring mol­ecules to form chains, which are linked by *π*–*π* inter­actions, further stabilizing the crystal structures. The chains are linked *via* C—H⋯O hydrogen bonds, forming layers. This situation is similar to that in the recently reported structures of (*E*)-*N*′-benzyl­idene-4-chloro­benzene­sulfono­lydrazine and the 2-methyl­benzil­idene derivative, (*E*)-*N′*-(2-methyl­benzyl­idene)-4-chloro­benzene­sulfono­lydrazine (Salian *et al.*, 2018 ▸).

## Synthesis and crystallization   

**Synthesis of 4-nitro­benzene­sulfono­hydrazide**

Hydrazine hydrate (99%, 5 ml) was added to 4-nitro­benzene­sulfonyl chloride (0.01 mol), dissolved in ethanol (30 ml) at 273 K under constant stirring. The stirring continued for 15 min at 273 K and then at 303 K for 3 h. The reaction mixture was then concentrated by evaporating off the excess ethanol. The solid product obtained, *i.e.* 4-nitro­benzene­sulfono­hydrazide, was washed with cold water and dried.

**Synthesis of the title compounds (I), (II) and (III)**

The title compounds were synthesized by refluxing the mixtures of 4-nitro­benzene­sulfono­hydrazide (0.01 mol) and 0.01 mol of 2-chloro­benzaldehyde for (I), 2-methyl­benzaldehyde for (II), and 4-methyl­benzaldehyde for (III), in ethanol (30 ml) and two drops of glacial acetic acid for 4 h. The reaction mixtures were cooled to room temperature and concentrated by evaporating off the excess of solvent. The solid products obtained were washed with cold water, dried and recrystallized to constant melting points from ethanol. Purity of the compounds was checked by TLC. All three compounds were characterized by measuring their IR, ^1^H and ^13^C NMR spectra.

(***E*** **)-** ***N*** **′-(2-chloro­benzyl­idene)-4-nitro­benzene­sulfono­hydrazide (I)**

Colourless prismatic crystals; m.p: 438–439 K; IR (cm^−1^): 3182.5 (N—H *asym. stretch*), 1604.8 (C=N), 1311.6 (S=O *asym. stretch*) and 1168.9 (S=O *sym. stretch*).

^1^H NMR (400 MHz, DMSO-*d_6_*): δ 7.16–7.18 (*m*, 2H, Ar-H), 7.45–7.47 (*m*, 2H, Ar-H), 7.91 (*s*, 1H), 8.15 (*d*, 2H, Ar-H), 8.41 (*d*, 2H, Ar-H), 11.71 (*s*, 1H): ^13^C NMR (400 MHz, DMSO-*d_6_*): δ 124.41, 126.67, 127.34, 128.75, 129.67, 130.55, 131.46, 133.08, 143.80, 144.27, 149.88.

**(** ***E*** **)-** ***N*** **′-(2-methyl­benzyl­idene)-4-nitro­benzene­sulfono­hydrazide (II)**

Yellow rod-shaped crystals; m.p: 417–418 K; IR (cm^−1^): 3217.3 (N—H *asym. stretch*), 1602.9 (C=N), 1332.1 (S=O *asym. stretch*) and 1172.7 (S=O *sym. stretch*).

^1^H NMR (400 MHz, DMSO-*d_6_*): δ 2.31 (*s*, 3H), 7.34-7.37 (*m*, 3H, Ar-H), 7.40 (*t*, 1H, Ar-H), 7.81 (*d*, 1H, Ar-H), 8.16 (*d*, 1H, Ar-H), 8.31 (*s*, 1H), 8.44 (*d*, 2H, Ar-H), 12.10 (*s*, 1H). ^13^C NMR (400 MHz, DMSO-*d_6_*): δ 20.97, 124.21, 125.90, 126.78, 128.75, 129.01, 130.61, 131.68, 139.99, 144.48, 148.13, 149.74.

(***E*** **)-** ***N*** **′-(4-methyl­benzyl­idene)-4-nitro­benzene­sulfono­hydrazide (III)**

Yellow prismatic crystals; m.p: 447–448 K; IR (cm^−1^): 3291.5 (N—H *asym. stretch*), 1606.7 (C=N), 1305.8 (S=O *asym. stretch*) and 1165.0 (S=O *sym. stretch*).

^1^H NMR (400 MHz, DMSO-*d_6_*): δ 2.31 (*s*, 3H), 7.34 (*d*, 2H, Ar-H), 7.60 (*d*, 2H, Ar-H), 8.16 (*d*, 2H, Ar-H), 8.30 (*s*, 1H), 8.42 (*d*, 2H, Ar-H), 12.03 (*s*, 1H): ^13^C NMR (400 MHz, DMSO-*d_6_*): δ 20.96, 123.81, 126.65, 128.59, 128.96, 130.47, 139.94, 144.64, 147.96, 149.58.

Single crystals of the title compounds used for the single-crystal X-ray diffraction analyses were obtained by slow evaporation of the solvent in their DMF solutions at room temperature**.**

## Refinement   

Crystal data, data collection and structure refinement details are summarized in Table 5 ▸. The C-bound H atoms were positioned with idealized geometry and refined using a riding model with the aromatic C—H = 0.93 Å. The amino H atoms were refined with an N—H distance restraint of 0.86 (2) Å. For (III), the H atoms of the water mol­ecule were refined with the O—H distance restrained to 0.82 (2) Å. All H atoms were refined with isotropic displacement parameters set at 1.2*U*_eq_(C-aromatic, N, O) and 1.5*U*_eq_(C-meth­yl). For (I), the low angle reflection (0 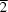 1) had a poor agreement with its calculated value and was omitted from the refinement.

## Supplementary Material

Crystal structure: contains datablock(s) I, II, III, global. DOI: 10.1107/S2056989018015207/su5460sup1.cif

Structure factors: contains datablock(s) I. DOI: 10.1107/S2056989018015207/su5460Isup2.hkl

Structure factors: contains datablock(s) II. DOI: 10.1107/S2056989018015207/su5460IIsup3.hkl

Structure factors: contains datablock(s) III. DOI: 10.1107/S2056989018015207/su5460IIIsup4.hkl

Supporting information file. DOI: 10.1107/S2056989018015207/su5460Isup5.cml

Supporting information file. DOI: 10.1107/S2056989018015207/su5460IIsup6.cml

Supporting information file. DOI: 10.1107/S2056989018015207/su5460IIIsup7.cml

CCDC references: 1578712, 1578713, 1578715

Additional supporting information:  crystallographic information; 3D view; checkCIF report

## Figures and Tables

**Figure 2:**
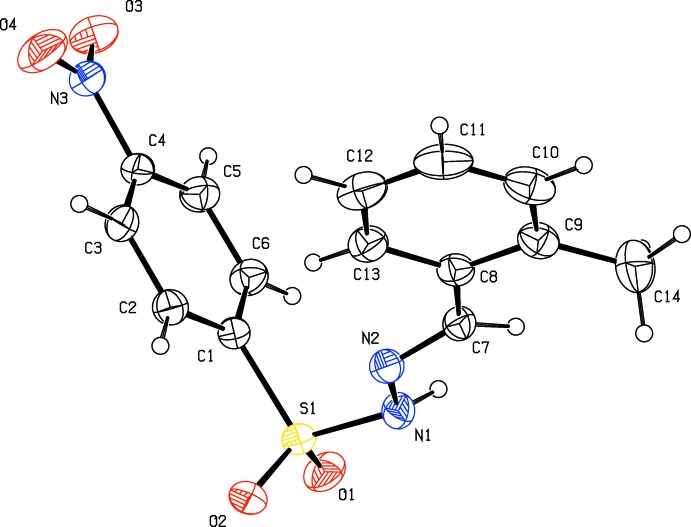
Mol­ecular structure of compound (II), showing the atom labelling and displacement ellipsoids drawn at the 30% probability level.

**Figure 3:**
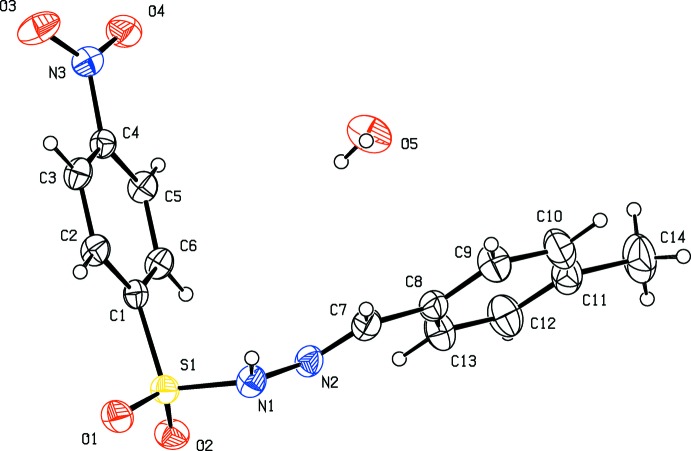
Mol­ecular structure of compound (III), showing the atom labelling and displacement ellipsoids drawn at the 30% probability level.

**Table 1:** Hydrogen-bond geometry (Å, °) for (I)

*D*—H⋯*A*	*D*—H	H⋯*A*	*D*⋯*A*	*D*—H⋯*A*
N1—H1*N*⋯O1^i^	0.86 (2)	2.02 (3)	2.853 (4)	163 (4)
C3—H3⋯O2^ii^	0.93	2.46	3.290 (4)	149

Symmetry codes: (i) 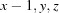; (ii) 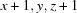.

**Table 2:** Hydrogen-bond geometry (Å, °) for (II)

*D*—H⋯*A*	*D*—H	H⋯*A*	*D*⋯*A*	*D*—H⋯*A*
N1—H1*N*⋯O3^i^	0.84 (2)	2.51 (2)	3.230 (2)	144 (2)
N1—H1*N*⋯O4^i^	0.84 (2)	2.44 (2)	3.260 (3)	164 (2)
C2—H2⋯O2^ii^	0.93	2.58	3.284 (2)	133
C12—H12⋯O1^iii^	0.93	2.44	3.341 (3)	164

Symmetry codes: (i) 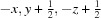; (ii) 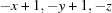; (iii) 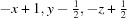.

**Table 3:** Hydrogen-bond geometry (Å, °) for (III)

*D*—H⋯*A*	*D*—H	H⋯*A*	*D*⋯*A*	*D*—H⋯*A*
N1—H1*N*⋯O5^i^	0.85 (2)	2.00 (2)	2.848 (2)	173 (2)
O5—H51⋯O2^ii^	0.81 (2)	2.29 (2)	3.006 (2)	148 (3)
O5—H52⋯O1^iii^	0.80 (2)	2.17 (2)	2.949 (2)	166 (3)
C5—H5⋯O1^iii^	0.93	2.52	3.167 (2)	127

Symmetry codes: (i) 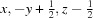; (ii) 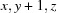; (iii) 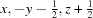.

**Table 4:** Hirshfeld contact inter­actions (%).

Contact type	(I)	(II)	(III)
O⋯H/H⋯O	35.0	37.3	32.0
H⋯H	17.5	28.4	37.2
C⋯H/H⋯C	17.3	13.4	11.0
O⋯C/C⋯O	4.3	1.8	9.4
C⋯C	1.0	7.8	0.3
N⋯H/H⋯N	4.3	7.3	5.0
N⋯C/C⋯N	2.2	0.1	1.2
O⋯N/N⋯O	1.1	1.4	1.4
O⋯O	1.9	2.4	0.0
S⋯C/C⋯S	0.0	0.1	0.1
Cl⋯C/C⋯Cl	4.7	-	-
Cl⋯H/H⋯Cl	6.1	-	-
Cl⋯O/O⋯Cl	3.1	-	-
Cl⋯N/N⋯Cl	1.4	-	-

**Table 5:** Experimental details

	(I)	(II)	(III)
Crystal data			
Chemical formula	C_13_H_10_ClN_3_O_4_S	C_14_H_13_N_3_O_4_S	C_14_H_13_N_3_O_4_S·H_2_O
*M*_r_	339.75	319.33	337.35
Crystal system, space group	Monoclinic, *P*2_1_	Monoclinic, *P*2_1_/*c*	Monoclinic, *P*2_1_/*c*
Temperature (K)	293	293	293
*a*, *b*, *c* (Å)	4.9498 (6), 22.340 (3), 7.0003 (9)	7.190 (1), 15.288 (2), 13.596 (2)	22.589 (2), 5.4424 (4), 12.7180 (9)
β (°)	104.40 (1)	97.68 (2)	92.146 (6)
*V* (Å^3^)	749.76 (17)	1481.1 (4)	1562.4 (2)
*Z*	2	4	4
Radiation type	Mo *K*α	Mo *K*α	Mo *K*α
μ (mm^−1^)	0.42	0.24	0.24
Crystal size (mm)	0.24 × 0.24 × 0.12	0.50 × 0.48 × 0.24	0.40 × 0.36 × 0.16

Data collection			
Diffractometer	Oxford Diffraction Xcalibur single crystal X-ray diffractometer with Sapphire CCD detector	Oxford Diffraction Xcalibur single crystal X-ray diffractometer with Sapphire CCD detector	Oxford Diffraction Xcalibur single crystal X-ray diffractometer with Sapphire CCD detector
Absorption correction	Multi-scan (*CrysAlis RED*; Oxford Diffraction, 2009 ▸)	Multi-scan (*CrysAlis RED*; Oxford Diffraction, 2009 ▸)	Multi-scan (*CrysAlis RED*; Oxford Diffraction, 2009 ▸)
*T*_min_, *T*_max_	0.907, 0.952	0.889, 0.945	0.911, 0.963
No. of measured, independent and observed [*I* > 2σ(*I*)] reflections	4582, 2696, 2457	9810, 2719, 2271	9384, 2870, 2178
*R*_int_	0.015	0.020	0.021
(sin θ/λ)_max_ (Å^−1^)	0.602	0.602	0.602

Refinement			
*R*[*F* ^2^ > 2σ(*F* ^2^)], *wR*(*F* ^2^), *S*	0.030, 0.078, 1.01	0.038, 0.097, 1.05	0.034, 0.092, 1.02
No. of reflections	2696	2719	2870
No. of parameters	202	203	218
No. of restraints	2	2	6
H-atom treatment	H atoms treated by a mixture of independent and constrained refinement	H atoms treated by a mixture of independent and constrained refinement	H atoms treated by a mixture of independent and constrained refinement
Δρ_max_, Δρ_min_ (e Å^−3^)	0.14, −0.20	0.28, −0.32	0.19, −0.28
Absolute structure	Flack *x* determined using 1102 quotients [(*I* ^+^)−(*I* ^−^)]/[(*I* ^+^)+(*I* ^−^)] (Parsons *et al.*, 2013 ▸)	–	–
Absolute structure parameter	0.05 (3)	–	–

Computer programs: *CrysAlis CCD* and *CrysAlis RED* (Oxford Diffraction, 2009 ▸), *SHELXS2013* (Sheldrick, 2008 ▸), *SHELXL2014* (Sheldrick, 2015 ▸) and *PLATON* (Spek, 2009 ▸).

## supplementary crystallographic information

### (E)-N'-(2-Chlorobenzylidene)-4-nitrobenzenesulfonohydrazide (I) Crystal data

**Table:**

C_13_H_10_ClN_3_O_4_S	*F*(000) = 348
*M**_r_* = 339.75	*D*_x_ = 1.505 Mg m^−^^3^
Monoclinic, *P*2_1_	Mo *K*α radiation, λ = 0.71073 Å
*a* = 4.9498 (6) Å	Cell parameters from 1264 reflections
*b* = 22.340 (3) Å	θ = 3.0–27.7°
*c* = 7.0003 (9) Å	µ = 0.42 mm^−^^1^
β = 104.40 (1)°	*T* = 293 K
*V* = 749.76 (17) Å^3^	Prism, colourless
*Z* = 2	0.24 × 0.24 × 0.12 mm

### (E)-N'-(2-Chlorobenzylidene)-4-nitrobenzenesulfonohydrazide (I) Data collection

**Table:**

Oxford Diffraction Xcalibur single crystal X-ray diffractometer with Sapphire CCD detector	2457 reflections with *I* > 2σ(*I*)
Radiation source: Enhance (Mo) X-ray Source	*R*_int_ = 0.015
Rotation method data acquisition using ω scans.	θ_max_ = 25.4°, θ_min_ = 3.0°
Absorption correction: multi-scan (CrysAlis RED; Oxford Diffraction, 2009)	*h* = −3→5
*T*_min_ = 0.907, *T*_max_ = 0.952	*k* = −26→26
4582 measured reflections	*l* = −8→7
2696 independent reflections	

### (E)-N'-(2-Chlorobenzylidene)-4-nitrobenzenesulfonohydrazide (I) Refinement

**Table:**

Refinement on *F*^2^	Secondary atom site location: difference Fourier map
Least-squares matrix: full	Hydrogen site location: mixed
*R*[*F*^2^ > 2σ(*F*^2^)] = 0.030	H atoms treated by a mixture of independent and constrained refinement
*wR*(*F*^2^) = 0.078	*w* = 1/[σ^2^(*F*_o_^2^) + (0.0489*P*)^2^ + 0.0544*P*] where *P* = (*F*_o_^2^ + 2*F*_c_^2^)/3
*S* = 1.01	(Δ/σ)_max_ < 0.001
2696 reflections	Δρ_max_ = 0.14 e Å^−^^3^
202 parameters	Δρ_min_ = −0.20 e Å^−^^3^
2 restraints	Absolute structure: Flack *x* determined using 1102 quotients [(*I*^+^)-(*I*^-^)]/[(*I*^+^)+(*I*^-^)] (Parsons *et al.*, 2013)
Primary atom site location: structure-invariant direct methods	Absolute structure parameter: 0.05 (3)

### (E)-N'-(2-Chlorobenzylidene)-4-nitrobenzenesulfonohydrazide (I) Special details

**Table:**

Geometry. All esds (except the esd in the dihedral angle between two l.s. planes) are estimated using the full covariance matrix. The cell esds are taken into account individually in the estimation of esds in distances, angles and torsion angles; correlations between esds in cell parameters are only used when they are defined by crystal symmetry. An approximate (isotropic) treatment of cell esds is used for estimating esds involving l.s. planes.

### (E)-N'-(2-Chlorobenzylidene)-4-nitrobenzenesulfonohydrazide (I) Fractional atomic coordinates and isotropic or equivalent isotropic displacement parameters (Å^2^)

**Table:**

	*x*	*y*	*z*	*U*_iso_*/*U*_eq_	
Cl1	0.0897 (2)	−0.10843 (5)	0.44188 (16)	0.0737 (3)	
S1	0.59163 (16)	0.12968 (4)	0.08796 (12)	0.0429 (2)	
O1	0.8691 (5)	0.11233 (13)	0.0900 (4)	0.0603 (7)	
O2	0.4169 (5)	0.15582 (12)	−0.0845 (3)	0.0519 (6)	
O3	0.8117 (8)	0.27393 (16)	0.9402 (5)	0.0788 (9)	
O4	0.4147 (8)	0.3096 (2)	0.7925 (5)	0.1064 (14)	
N1	0.4239 (6)	0.07005 (13)	0.1288 (4)	0.0445 (7)	
H1N	0.248 (5)	0.0750 (18)	0.109 (5)	0.053*	
N2	0.5519 (6)	0.04007 (13)	0.3042 (4)	0.0472 (7)	
N3	0.6159 (8)	0.27887 (16)	0.7968 (5)	0.0619 (9)	
C1	0.6093 (7)	0.17729 (15)	0.2929 (5)	0.0411 (7)	
C2	0.8188 (7)	0.16808 (16)	0.4642 (5)	0.0480 (8)	
H2	0.9556	0.1393	0.4672	0.058*	
C3	0.8216 (7)	0.20189 (17)	0.6288 (5)	0.0509 (9)	
H3	0.9590	0.1963	0.7451	0.061*	
C4	0.6175 (8)	0.24394 (16)	0.6174 (5)	0.0467 (8)	
C5	0.4109 (8)	0.25443 (17)	0.4481 (6)	0.0517 (9)	
H5	0.2762	0.2836	0.4456	0.062*	
C6	0.4083 (8)	0.22071 (16)	0.2829 (5)	0.0479 (8)	
H6	0.2728	0.2271	0.1662	0.057*	
C7	0.3937 (8)	0.00577 (16)	0.3719 (6)	0.0498 (9)	
H7	0.2072	0.0013	0.3054	0.060*	
C8	0.5085 (8)	−0.02677 (16)	0.5566 (5)	0.0504 (9)	
C9	0.3835 (9)	−0.07856 (17)	0.6049 (6)	0.0553 (9)	
C10	0.4901 (10)	−0.1087 (2)	0.7781 (6)	0.0719 (11)	
H10	0.4014	−0.1428	0.8084	0.086*	
C11	0.7282 (13)	−0.0882 (2)	0.9061 (7)	0.0875 (17)	
H11	0.8007	−0.1084	1.0237	0.105*	
C12	0.8616 (12)	−0.0377 (2)	0.8620 (7)	0.0861 (16)	
H12	1.0268	−0.0247	0.9471	0.103*	
C13	0.7476 (11)	−0.00665 (19)	0.6908 (6)	0.0715 (13)	
H13	0.8324	0.0285	0.6645	0.086*	

### (E)-N'-(2-Chlorobenzylidene)-4-nitrobenzenesulfonohydrazide (I) Atomic displacement parameters (Å^2^)

**Table:**

	*U*^11^	*U*^22^	*U*^33^	*U*^12^	*U*^13^	*U*^23^
Cl1	0.0766 (7)	0.0678 (6)	0.0675 (6)	−0.0191 (6)	0.0002 (5)	0.0046 (6)
S1	0.0340 (4)	0.0534 (5)	0.0430 (4)	0.0058 (4)	0.0128 (3)	0.0063 (4)
O1	0.0368 (13)	0.080 (2)	0.0683 (16)	0.0089 (12)	0.0213 (12)	−0.0014 (14)
O2	0.0519 (14)	0.0638 (15)	0.0395 (13)	0.0067 (12)	0.0103 (11)	0.0142 (12)
O3	0.084 (2)	0.083 (2)	0.0589 (18)	−0.0005 (18)	−0.0023 (17)	−0.0094 (17)
O4	0.110 (3)	0.128 (3)	0.076 (2)	0.055 (3)	0.011 (2)	−0.027 (2)
N1	0.0362 (15)	0.0500 (16)	0.0443 (16)	0.0064 (13)	0.0041 (13)	0.0079 (12)
N2	0.0466 (17)	0.0437 (16)	0.0472 (17)	0.0088 (14)	0.0039 (14)	0.0061 (13)
N3	0.072 (2)	0.0595 (19)	0.053 (2)	0.0030 (18)	0.0142 (19)	−0.0050 (17)
C1	0.0350 (17)	0.0456 (17)	0.0433 (18)	0.0022 (14)	0.0110 (14)	0.0077 (14)
C2	0.0355 (18)	0.054 (2)	0.052 (2)	0.0097 (15)	0.0059 (16)	0.0040 (16)
C3	0.0401 (19)	0.059 (2)	0.048 (2)	0.0028 (16)	0.0006 (16)	0.0039 (17)
C4	0.048 (2)	0.047 (2)	0.045 (2)	−0.0013 (16)	0.0096 (17)	0.0016 (16)
C5	0.048 (2)	0.050 (2)	0.056 (2)	0.0108 (16)	0.0100 (19)	0.0057 (18)
C6	0.044 (2)	0.052 (2)	0.0443 (19)	0.0118 (16)	0.0048 (16)	0.0082 (16)
C7	0.057 (2)	0.046 (2)	0.043 (2)	0.0017 (16)	0.0059 (17)	−0.0016 (16)
C8	0.065 (2)	0.0430 (19)	0.0394 (18)	0.0024 (18)	0.0053 (17)	−0.0017 (15)
C9	0.070 (3)	0.0474 (19)	0.0442 (19)	−0.0012 (18)	0.0055 (18)	−0.0009 (16)
C10	0.101 (3)	0.058 (2)	0.051 (2)	−0.007 (3)	0.007 (2)	0.007 (2)
C11	0.132 (5)	0.068 (3)	0.046 (2)	−0.005 (3)	−0.011 (3)	0.011 (2)
C12	0.111 (4)	0.074 (3)	0.051 (2)	−0.015 (3)	−0.021 (2)	0.000 (2)
C13	0.095 (4)	0.054 (2)	0.052 (3)	−0.018 (2)	−0.006 (2)	0.003 (2)

### (E)-N'-(2-Chlorobenzylidene)-4-nitrobenzenesulfonohydrazide (I) Geometric parameters (Å, º)

**Table:**

Cl1—C9	1.743 (4)	C4—C5	1.379 (5)
S1—O1	1.424 (3)	C5—C6	1.378 (5)
S1—O2	1.424 (2)	C5—H5	0.9300
S1—N1	1.632 (3)	C6—H6	0.9300
S1—C1	1.770 (3)	C7—C8	1.468 (5)
O3—N3	1.214 (4)	C7—H7	0.9300
O4—N3	1.204 (4)	C8—C13	1.390 (6)
N1—N2	1.404 (4)	C8—C9	1.393 (5)
N1—H1N	0.86 (2)	C9—C10	1.373 (6)
N2—C7	1.269 (5)	C10—C11	1.370 (7)
N3—C4	1.481 (5)	C10—H10	0.9300
C1—C6	1.379 (5)	C11—C12	1.380 (7)
C1—C2	1.391 (5)	C11—H11	0.9300
C2—C3	1.375 (5)	C12—C13	1.378 (6)
C2—H2	0.9300	C12—H12	0.9300
C3—C4	1.367 (5)	C13—H13	0.9300
C3—H3	0.9300		
			
O1—S1—O2	120.09 (15)	C6—C5—H5	120.7
O1—S1—N1	107.89 (16)	C4—C5—H5	120.7
O2—S1—N1	104.77 (15)	C1—C6—C5	119.1 (3)
O1—S1—C1	107.59 (16)	C1—C6—H6	120.5
O2—S1—C1	109.75 (16)	C5—C6—H6	120.5
N1—S1—C1	105.87 (15)	N2—C7—C8	119.2 (3)
N2—N1—S1	113.8 (2)	N2—C7—H7	120.4
N2—N1—H1N	115 (3)	C8—C7—H7	120.4
S1—N1—H1N	114 (3)	C13—C8—C9	117.3 (3)
C7—N2—N1	115.4 (3)	C13—C8—C7	120.9 (4)
O4—N3—O3	123.9 (4)	C9—C8—C7	121.8 (3)
O4—N3—C4	117.3 (4)	C10—C9—C8	121.8 (4)
O3—N3—C4	118.8 (4)	C10—C9—Cl1	117.7 (3)
C6—C1—C2	121.4 (3)	C8—C9—Cl1	120.5 (3)
C6—C1—S1	119.4 (3)	C11—C10—C9	119.4 (4)
C2—C1—S1	119.0 (3)	C11—C10—H10	120.3
C3—C2—C1	119.4 (3)	C9—C10—H10	120.3
C3—C2—H2	120.3	C10—C11—C12	120.7 (4)
C1—C2—H2	120.3	C10—C11—H11	119.7
C4—C3—C2	118.3 (3)	C12—C11—H11	119.7
C4—C3—H3	120.8	C11—C12—C13	119.3 (5)
C2—C3—H3	120.8	C11—C12—H12	120.3
C3—C4—C5	123.1 (3)	C13—C12—H12	120.3
C3—C4—N3	118.2 (3)	C12—C13—C8	121.4 (4)
C5—C4—N3	118.7 (3)	C12—C13—H13	119.3
C6—C5—C4	118.6 (3)	C8—C13—H13	119.3
			
O1—S1—N1—N2	56.0 (3)	C3—C4—C5—C6	0.5 (6)
O2—S1—N1—N2	−175.0 (2)	N3—C4—C5—C6	−178.2 (3)
C1—S1—N1—N2	−59.0 (3)	C2—C1—C6—C5	−1.9 (5)
S1—N1—N2—C7	159.3 (3)	S1—C1—C6—C5	174.9 (3)
O1—S1—C1—C6	151.0 (3)	C4—C5—C6—C1	0.8 (5)
O2—S1—C1—C6	18.7 (3)	N1—N2—C7—C8	−179.1 (3)
N1—S1—C1—C6	−93.9 (3)	N2—C7—C8—C13	22.2 (5)
O1—S1—C1—C2	−32.1 (3)	N2—C7—C8—C9	−158.0 (4)
O2—S1—C1—C2	−164.4 (3)	C13—C8—C9—C10	0.2 (6)
N1—S1—C1—C2	83.0 (3)	C7—C8—C9—C10	−179.6 (4)
C6—C1—C2—C3	1.7 (5)	C13—C8—C9—Cl1	−177.9 (3)
S1—C1—C2—C3	−175.1 (3)	C7—C8—C9—Cl1	2.3 (5)
C1—C2—C3—C4	−0.4 (5)	C8—C9—C10—C11	−1.1 (7)
C2—C3—C4—C5	−0.7 (6)	Cl1—C9—C10—C11	177.1 (4)
C2—C3—C4—N3	178.0 (3)	C9—C10—C11—C12	−0.2 (8)
O4—N3—C4—C3	−170.0 (4)	C10—C11—C12—C13	2.3 (9)
O3—N3—C4—C3	8.2 (5)	C11—C12—C13—C8	−3.2 (9)
O4—N3—C4—C5	8.7 (6)	C9—C8—C13—C12	2.0 (7)
O3—N3—C4—C5	−173.0 (4)	C7—C8—C13—C12	−178.2 (5)

### (E)-N'-(2-Chlorobenzylidene)-4-nitrobenzenesulfonohydrazide (I) Hydrogen-bond geometry (Å, º)

**Table:**

*D*—H···*A*	*D*—H	H···*A*	*D*···*A*	*D*—H···*A*
N1—H1*N*···O1^i^	0.86 (2)	2.02 (3)	2.853 (4)	163 (4)
C3—H3···O2^ii^	0.93	2.46	3.290 (4)	149

Symmetry codes: (i) *x*−1, *y*, *z*; (ii) *x*+1, *y*, *z*+1.

### (E)-N'-(2-Methylbenzylidene)-4-nitrobenzenesulfonohydrazide (II) Crystal data

**Table:**

C_14_H_13_N_3_O_4_S	*F*(000) = 664
*M**_r_* = 319.33	*D*_x_ = 1.432 Mg m^−^^3^
Monoclinic, *P*2_1_/*c*	Mo *K*α radiation, λ = 0.71073 Å
*a* = 7.190 (1) Å	Cell parameters from 2833 reflections
*b* = 15.288 (2) Å	θ = 2.6–27.8°
*c* = 13.596 (2) Å	µ = 0.24 mm^−^^1^
β = 97.68 (2)°	*T* = 293 K
*V* = 1481.1 (4) Å^3^	Rod, yellow
*Z* = 4	0.50 × 0.48 × 0.24 mm

### (E)-N'-(2-Methylbenzylidene)-4-nitrobenzenesulfonohydrazide (II) Data collection

**Table:**

Oxford Diffraction Xcalibur single crystal X-ray diffractometer with Sapphire CCD detector	2271 reflections with *I* > 2σ(*I*)
Radiation source: Enhance (Mo) X-ray Source	*R*_int_ = 0.020
Rotation method data acquisition using ω scans.	θ_max_ = 25.4°, θ_min_ = 2.7°
Absorption correction: multi-scan (CrysAlis RED; Oxford Diffraction, 2009)	*h* = −8→5
*T*_min_ = 0.889, *T*_max_ = 0.945	*k* = −18→18
9810 measured reflections	*l* = −11→16
2719 independent reflections	

### (E)-N'-(2-Methylbenzylidene)-4-nitrobenzenesulfonohydrazide (II) Refinement

**Table:**

Refinement on *F*^2^	Primary atom site location: structure-invariant direct methods
Least-squares matrix: full	Secondary atom site location: difference Fourier map
*R*[*F*^2^ > 2σ(*F*^2^)] = 0.038	Hydrogen site location: mixed
*wR*(*F*^2^) = 0.097	H atoms treated by a mixture of independent and constrained refinement
*S* = 1.05	*w* = 1/[σ^2^(*F*_o_^2^) + (0.0372*P*)^2^ + 0.8005*P*] where *P* = (*F*_o_^2^ + 2*F*_c_^2^)/3
2719 reflections	(Δ/σ)_max_ = 0.001
203 parameters	Δρ_max_ = 0.28 e Å^−^^3^
2 restraints	Δρ_min_ = −0.32 e Å^−^^3^

### (E)-N'-(2-Methylbenzylidene)-4-nitrobenzenesulfonohydrazide (II) Special details

**Table:**

Geometry. All esds (except the esd in the dihedral angle between two l.s. planes) are estimated using the full covariance matrix. The cell esds are taken into account individually in the estimation of esds in distances, angles and torsion angles; correlations between esds in cell parameters are only used when they are defined by crystal symmetry. An approximate (isotropic) treatment of cell esds is used for estimating esds involving l.s. planes.

### (E)-N'-(2-Methylbenzylidene)-4-nitrobenzenesulfonohydrazide (II) Fractional atomic coordinates and isotropic or equivalent isotropic displacement parameters (Å^2^)

**Table:**

	*x*	*y*	*z*	*U*_iso_*/*U*_eq_	
C1	0.1811 (3)	0.53913 (12)	0.11782 (14)	0.0392 (4)	
C2	0.2549 (3)	0.45557 (13)	0.12179 (16)	0.0472 (5)	
H2	0.3779	0.4459	0.1104	0.057*	
C3	0.1428 (3)	0.38663 (13)	0.14295 (16)	0.0515 (5)	
H3	0.1894	0.3298	0.1470	0.062*	
C4	−0.0386 (3)	0.40354 (13)	0.15791 (14)	0.0458 (5)	
C5	−0.1143 (3)	0.48574 (14)	0.15301 (16)	0.0506 (5)	
H5	−0.2384	0.4948	0.1628	0.061*	
C6	−0.0019 (3)	0.55474 (13)	0.13323 (16)	0.0475 (5)	
H6	−0.0489	0.6115	0.1303	0.057*	
C7	0.5612 (3)	0.62282 (12)	0.35371 (15)	0.0414 (4)	
H7	0.5251	0.6771	0.3756	0.050*	
C8	0.6602 (3)	0.56164 (13)	0.42534 (15)	0.0419 (5)	
C9	0.7375 (3)	0.58901 (15)	0.52053 (16)	0.0513 (5)	
C10	0.8215 (3)	0.52648 (19)	0.58632 (19)	0.0651 (7)	
H10	0.8717	0.5436	0.6500	0.078*	
C11	0.8321 (3)	0.4407 (2)	0.5599 (2)	0.0701 (8)	
H11	0.8900	0.4004	0.6053	0.084*	
C12	0.7574 (3)	0.41387 (16)	0.4665 (2)	0.0651 (7)	
H12	0.7657	0.3555	0.4483	0.078*	
C13	0.6700 (3)	0.47378 (14)	0.39960 (18)	0.0513 (5)	
H13	0.6172	0.4553	0.3369	0.062*	
C14	0.7337 (4)	0.68279 (18)	0.5534 (2)	0.0781 (8)	
H14A	0.6070	0.7041	0.5427	0.117*	
H14B	0.8104	0.7174	0.5157	0.117*	
H14C	0.7813	0.6868	0.6226	0.117*	
N1	0.4195 (2)	0.66562 (10)	0.20225 (12)	0.0431 (4)	
H1N	0.347 (3)	0.6988 (13)	0.2284 (16)	0.052*	
N2	0.5234 (2)	0.60339 (10)	0.26201 (12)	0.0413 (4)	
N3	−0.1566 (3)	0.32996 (13)	0.18268 (14)	0.0592 (5)	
O1	0.2086 (2)	0.69660 (9)	0.05038 (11)	0.0591 (4)	
O2	0.4735 (2)	0.59482 (10)	0.04571 (11)	0.0590 (4)	
O3	−0.3229 (3)	0.34312 (13)	0.18613 (15)	0.0833 (6)	
O4	−0.0808 (3)	0.25947 (12)	0.19986 (16)	0.0867 (6)	
S1	0.32586 (7)	0.62811 (3)	0.09402 (4)	0.04312 (16)	

### (E)-N'-(2-Methylbenzylidene)-4-nitrobenzenesulfonohydrazide (II) Atomic displacement parameters (Å^2^)

**Table:**

	*U*^11^	*U*^22^	*U*^33^	*U*^12^	*U*^13^	*U*^23^
C1	0.0478 (11)	0.0349 (10)	0.0357 (10)	−0.0027 (8)	0.0079 (8)	0.0008 (8)
C2	0.0516 (12)	0.0409 (11)	0.0520 (12)	0.0030 (9)	0.0168 (10)	−0.0018 (9)
C3	0.0726 (14)	0.0318 (10)	0.0524 (13)	−0.0001 (10)	0.0170 (11)	−0.0017 (9)
C4	0.0584 (12)	0.0446 (11)	0.0351 (10)	−0.0133 (9)	0.0086 (9)	−0.0002 (8)
C5	0.0455 (11)	0.0544 (13)	0.0526 (13)	−0.0045 (9)	0.0095 (9)	0.0029 (10)
C6	0.0487 (12)	0.0400 (11)	0.0542 (13)	0.0035 (9)	0.0088 (10)	0.0048 (9)
C7	0.0414 (10)	0.0371 (10)	0.0472 (11)	0.0000 (8)	0.0116 (8)	−0.0037 (9)
C8	0.0340 (10)	0.0463 (11)	0.0473 (12)	0.0009 (8)	0.0121 (8)	0.0023 (9)
C9	0.0409 (11)	0.0646 (14)	0.0494 (13)	−0.0001 (10)	0.0097 (9)	0.0019 (11)
C10	0.0462 (13)	0.096 (2)	0.0527 (14)	0.0022 (13)	0.0074 (10)	0.0156 (14)
C11	0.0456 (13)	0.085 (2)	0.0818 (19)	0.0105 (12)	0.0173 (13)	0.0388 (16)
C12	0.0535 (13)	0.0495 (13)	0.097 (2)	0.0086 (11)	0.0280 (13)	0.0170 (13)
C13	0.0481 (12)	0.0456 (12)	0.0628 (14)	0.0025 (9)	0.0172 (10)	0.0021 (10)
C14	0.0869 (19)	0.0817 (19)	0.0621 (17)	−0.0032 (15)	−0.0039 (14)	−0.0177 (14)
N1	0.0500 (10)	0.0349 (9)	0.0449 (10)	0.0041 (7)	0.0077 (8)	−0.0020 (7)
N2	0.0413 (9)	0.0383 (9)	0.0457 (10)	0.0024 (7)	0.0103 (7)	−0.0003 (7)
N3	0.0799 (11)	0.0527 (12)	0.0460 (10)	−0.0222 (10)	0.0126 (10)	−0.0024 (9)
O1	0.0738 (10)	0.0416 (8)	0.0592 (10)	−0.0047 (7)	−0.0012 (8)	0.0139 (7)
O2	0.0715 (10)	0.0577 (9)	0.0545 (9)	−0.0116 (8)	0.0326 (8)	−0.0072 (7)
O3	0.0790 (10)	0.0840 (13)	0.0928 (14)	−0.0339 (10)	0.0331 (11)	−0.0027 (11)
O4	0.1088 (16)	0.0507 (11)	0.1012 (15)	−0.0198 (10)	0.0163 (12)	0.0168 (10)
S1	0.0545 (3)	0.0361 (3)	0.0402 (3)	−0.0054 (2)	0.0117 (2)	0.0023 (2)

### (E)-N'-(2-Methylbenzylidene)-4-nitrobenzenesulfonohydrazide (II) Geometric parameters (Å, º)

**Table:**

C1—C6	1.381 (3)	C9—C14	1.503 (3)
C1—C2	1.381 (3)	C10—C11	1.364 (4)
C1—S1	1.7687 (19)	C10—H10	0.9300
C2—C3	1.380 (3)	C11—C12	1.375 (4)
C2—H2	0.9300	C11—H11	0.9300
C3—C4	1.371 (3)	C12—C13	1.381 (3)
C3—H3	0.9300	C12—H12	0.9300
C4—C5	1.368 (3)	C13—H13	0.9300
C4—N3	1.475 (3)	C14—H14A	0.9600
C5—C6	1.377 (3)	C14—H14B	0.9600
C5—H5	0.9300	C14—H14C	0.9600
C6—H6	0.9300	N1—N2	1.400 (2)
C7—N2	1.275 (2)	N1—S1	1.6380 (17)
C7—C8	1.465 (3)	N1—H1N	0.839 (15)
C7—H7	0.9300	N3—O4	1.216 (3)
C8—C13	1.392 (3)	N3—O3	1.220 (3)
C8—C9	1.402 (3)	O1—S1	1.4233 (15)
C9—C10	1.391 (3)	O2—S1	1.4157 (15)
			
C6—C1—C2	121.50 (18)	C9—C10—H10	119.1
C6—C1—S1	119.39 (15)	C10—C11—C12	120.1 (2)
C2—C1—S1	119.10 (15)	C10—C11—H11	120.0
C3—C2—C1	118.86 (19)	C12—C11—H11	120.0
C3—C2—H2	120.6	C11—C12—C13	119.8 (2)
C1—C2—H2	120.6	C11—C12—H12	120.1
C4—C3—C2	118.70 (19)	C13—C12—H12	120.1
C4—C3—H3	120.6	C12—C13—C8	120.5 (2)
C2—C3—H3	120.6	C12—C13—H13	119.7
C5—C4—C3	123.12 (19)	C8—C13—H13	119.7
C5—C4—N3	118.3 (2)	C9—C14—H14A	109.5
C3—C4—N3	118.5 (2)	C9—C14—H14B	109.5
C4—C5—C6	118.25 (19)	H14A—C14—H14B	109.5
C4—C5—H5	120.9	C9—C14—H14C	109.5
C6—C5—H5	120.9	H14A—C14—H14C	109.5
C5—C6—C1	119.56 (19)	H14B—C14—H14C	109.5
C5—C6—H6	120.2	N2—N1—S1	114.00 (12)
C1—C6—H6	120.2	N2—N1—H1N	118.6 (15)
N2—C7—C8	121.51 (18)	S1—N1—H1N	112.8 (15)
N2—C7—H7	119.2	C7—N2—N1	115.88 (16)
C8—C7—H7	119.2	O4—N3—O3	123.7 (2)
C13—C8—C9	119.5 (2)	O4—N3—C4	117.6 (2)
C13—C8—C7	119.03 (19)	O3—N3—C4	118.7 (2)
C9—C8—C7	121.37 (19)	O2—S1—O1	120.69 (10)
C10—C9—C8	118.2 (2)	O2—S1—N1	107.39 (9)
C10—C9—C14	119.2 (2)	O1—S1—N1	105.46 (9)
C8—C9—C14	122.5 (2)	O2—S1—C1	107.72 (9)
C11—C10—C9	121.8 (2)	O1—S1—C1	108.17 (9)
C11—C10—H10	119.1	N1—S1—C1	106.63 (9)
			
C6—C1—C2—C3	−0.9 (3)	C10—C11—C12—C13	−0.6 (3)
S1—C1—C2—C3	178.33 (16)	C11—C12—C13—C8	1.3 (3)
C1—C2—C3—C4	0.9 (3)	C9—C8—C13—C12	−1.0 (3)
C2—C3—C4—C5	−0.1 (3)	C7—C8—C13—C12	−178.05 (19)
C2—C3—C4—N3	−178.67 (19)	C8—C7—N2—N1	176.70 (15)
C3—C4—C5—C6	−0.8 (3)	S1—N1—N2—C7	−164.16 (14)
N3—C4—C5—C6	177.81 (19)	C5—C4—N3—O4	−170.4 (2)
C4—C5—C6—C1	0.8 (3)	C3—C4—N3—O4	8.3 (3)
C2—C1—C6—C5	0.0 (3)	C5—C4—N3—O3	8.7 (3)
S1—C1—C6—C5	−179.19 (16)	C3—C4—N3—O3	−172.7 (2)
N2—C7—C8—C13	−17.0 (3)	N2—N1—S1—O2	−57.23 (15)
N2—C7—C8—C9	165.96 (18)	N2—N1—S1—O1	172.86 (13)
C13—C8—C9—C10	−0.1 (3)	N2—N1—S1—C1	58.01 (15)
C7—C8—C9—C10	176.89 (18)	C6—C1—S1—O2	−158.52 (16)
C13—C8—C9—C14	179.5 (2)	C2—C1—S1—O2	22.28 (19)
C7—C8—C9—C14	−3.5 (3)	C6—C1—S1—O1	−26.54 (19)
C8—C9—C10—C11	0.9 (3)	C2—C1—S1—O1	154.26 (16)
C14—C9—C10—C11	−178.7 (2)	C6—C1—S1—N1	86.46 (17)
C9—C10—C11—C12	−0.5 (4)	C2—C1—S1—N1	−92.74 (17)

### (E)-N'-(2-Methylbenzylidene)-4-nitrobenzenesulfonohydrazide (II) Hydrogen-bond geometry (Å, º)

**Table:**

*D*—H···*A*	*D*—H	H···*A*	*D*···*A*	*D*—H···*A*
N1—H1*N*···O3^i^	0.84 (2)	2.51 (2)	3.230 (2)	144 (2)
N1—H1*N*···O4^i^	0.84 (2)	2.44 (2)	3.260 (3)	164 (2)
C2—H2···O2^ii^	0.93	2.58	3.284 (2)	133
C12—H12···O1^iii^	0.93	2.44	3.341 (3)	164

Symmetry codes: (i) −*x*, *y*+1/2, −*z*+1/2; (ii) −*x*+1, −*y*+1, −*z*; (iii) −*x*+1, *y*−1/2, −*z*+1/2.

### (E)-N'-(4-Methylbenzylidene)-4-nitrobenzenesulfonohydrazide monohydrate (III) Crystal data

**Table:**

C_14_H_13_N_3_O_4_S·H_2_O	*F*(000) = 704
*M**_r_* = 337.35	*D*_x_ = 1.434 Mg m^−^^3^
Monoclinic, *P*2_1_/*c*	Mo *K*α radiation, λ = 0.71073 Å
*a* = 22.589 (2) Å	Cell parameters from 3331 reflections
*b* = 5.4424 (4) Å	θ = 3.1–27.8°
*c* = 12.7180 (9) Å	µ = 0.24 mm^−^^1^
β = 92.146 (6)°	*T* = 293 K
*V* = 1562.4 (2) Å^3^	Prism, yellow
*Z* = 4	0.40 × 0.36 × 0.16 mm

### (E)-N'-(4-Methylbenzylidene)-4-nitrobenzenesulfonohydrazide monohydrate (III) Data collection

**Table:**

Oxford Diffraction Xcalibur single crystal X-ray diffractometer with Sapphire CCD detector	2178 reflections with *I* > 2σ(*I*)
Radiation source: Enhance (Mo) X-ray Source	*R*_int_ = 0.021
Rotation method data acquisition using ω scans.	θ_max_ = 25.4°, θ_min_ = 3.2°
Absorption correction: multi-scan (CrysAlis RED; Oxford Diffraction, 2009)	*h* = −19→27
*T*_min_ = 0.911, *T*_max_ = 0.963	*k* = −6→6
9384 measured reflections	*l* = −14→15
2870 independent reflections	

### (E)-N'-(4-Methylbenzylidene)-4-nitrobenzenesulfonohydrazide monohydrate (III) Refinement

**Table:**

Refinement on *F*^2^	Primary atom site location: structure-invariant direct methods
Least-squares matrix: full	Secondary atom site location: difference Fourier map
*R*[*F*^2^ > 2σ(*F*^2^)] = 0.034	Hydrogen site location: mixed
*wR*(*F*^2^) = 0.092	H atoms treated by a mixture of independent and constrained refinement
*S* = 1.02	*w* = 1/[σ^2^(*F*_o_^2^) + (0.0464*P*)^2^ + 0.3615*P*] where *P* = (*F*_o_^2^ + 2*F*_c_^2^)/3
2870 reflections	(Δ/σ)_max_ < 0.001
218 parameters	Δρ_max_ = 0.19 e Å^−^^3^
6 restraints	Δρ_min_ = −0.28 e Å^−^^3^

### (E)-N'-(4-Methylbenzylidene)-4-nitrobenzenesulfonohydrazide monohydrate (III) Special details

**Table:**

Geometry. All esds (except the esd in the dihedral angle between two l.s. planes) are estimated using the full covariance matrix. The cell esds are taken into account individually in the estimation of esds in distances, angles and torsion angles; correlations between esds in cell parameters are only used when they are defined by crystal symmetry. An approximate (isotropic) treatment of cell esds is used for estimating esds involving l.s. planes.

### (E)-N'-(4-Methylbenzylidene)-4-nitrobenzenesulfonohydrazide monohydrate (III) Fractional atomic coordinates and isotropic or equivalent isotropic displacement parameters (Å^2^)

**Table:**

	*x*	*y*	*z*	*U*_iso_*/*U*_eq_	
S1	0.29814 (2)	−0.41337 (9)	0.00460 (3)	0.04151 (15)	
O1	0.31514 (6)	−0.4922 (3)	−0.09707 (10)	0.0540 (4)	
O2	0.28665 (6)	−0.5896 (2)	0.08395 (10)	0.0533 (4)	
O3	0.50400 (7)	0.4229 (3)	0.11440 (12)	0.0697 (5)	
O4	0.45497 (7)	0.4367 (3)	0.25661 (11)	0.0619 (4)	
N1	0.23739 (7)	−0.2563 (3)	−0.01813 (12)	0.0465 (4)	
H1N	0.2396 (9)	−0.153 (3)	−0.0681 (14)	0.056*	
N2	0.21027 (7)	−0.1771 (3)	0.07293 (12)	0.0470 (4)	
N3	0.46427 (7)	0.3533 (3)	0.16974 (13)	0.0463 (4)	
C1	0.35122 (7)	−0.2010 (3)	0.05425 (13)	0.0367 (4)	
C2	0.38690 (8)	−0.0791 (4)	−0.01472 (14)	0.0439 (4)	
H2	0.3852	−0.1181	−0.0860	0.053*	
C3	0.42513 (8)	0.1012 (4)	0.02325 (14)	0.0440 (4)	
H3	0.4500	0.1839	−0.0215	0.053*	
C4	0.42543 (7)	0.1550 (3)	0.12892 (14)	0.0383 (4)	
C5	0.39051 (8)	0.0344 (4)	0.19866 (14)	0.0431 (4)	
H5	0.3923	0.0746	0.2698	0.052*	
C6	0.35286 (8)	−0.1470 (3)	0.16104 (13)	0.0424 (4)	
H6	0.3289	−0.2321	0.2065	0.051*	
C7	0.17972 (8)	0.0167 (4)	0.06417 (16)	0.0505 (5)	
H7	0.1791	0.1035	0.0012	0.061*	
C8	0.14529 (8)	0.1070 (4)	0.15155 (16)	0.0501 (5)	
C9	0.11387 (10)	0.3238 (4)	0.14097 (19)	0.0669 (6)	
H9	0.1164	0.4165	0.0799	0.080*	
C10	0.07863 (11)	0.4042 (5)	0.2206 (2)	0.0754 (7)	
H10	0.0578	0.5505	0.2118	0.090*	
C11	0.07354 (10)	0.2750 (5)	0.31180 (19)	0.0670 (6)	
C12	0.10618 (11)	0.0627 (5)	0.32272 (19)	0.0725 (7)	
H12	0.1044	−0.0267	0.3848	0.087*	
C13	0.14130 (10)	−0.0210 (4)	0.24460 (17)	0.0626 (6)	
H13	0.1627	−0.1658	0.2545	0.075*	
C14	0.03449 (12)	0.3685 (6)	0.3974 (2)	0.0970 (10)	
H14A	0.0205	0.2322	0.4374	0.146*	
H14B	0.0013	0.4553	0.3660	0.146*	
H14C	0.0569	0.4774	0.4430	0.146*	
O5	0.25535 (9)	0.4283 (3)	0.31149 (13)	0.0753 (5)	
H51	0.2502 (12)	0.445 (5)	0.2487 (14)	0.090*	
H52	0.2685 (12)	0.296 (4)	0.328 (2)	0.090*	

### (E)-N'-(4-Methylbenzylidene)-4-nitrobenzenesulfonohydrazide monohydrate (III) Atomic displacement parameters (Å^2^)

**Table:**

	*U*^11^	*U*^22^	*U*^33^	*U*^12^	*U*^13^	*U*^23^
S1	0.0456 (3)	0.0425 (3)	0.0370 (3)	−0.0023 (2)	0.00972 (18)	−0.0038 (2)
O1	0.0622 (9)	0.0571 (8)	0.0437 (7)	−0.0028 (7)	0.0164 (6)	−0.0144 (7)
O2	0.0653 (9)	0.0448 (7)	0.0506 (8)	−0.0067 (7)	0.0131 (7)	0.0041 (6)
O3	0.0700 (10)	0.0800 (11)	0.0592 (9)	−0.0336 (9)	0.0065 (8)	0.0081 (8)
O4	0.0664 (9)	0.0616 (9)	0.0579 (8)	−0.0062 (8)	0.0040 (7)	−0.0154 (7)
N1	0.0452 (9)	0.0583 (10)	0.0363 (9)	−0.0017 (8)	0.0052 (7)	−0.0027 (7)
N2	0.0416 (9)	0.0569 (10)	0.0430 (9)	−0.0024 (8)	0.0087 (7)	−0.0077 (8)
N3	0.0474 (9)	0.0467 (9)	0.0444 (8)	−0.0028 (7)	−0.0023 (7)	0.0059 (7)
C1	0.0349 (9)	0.0406 (9)	0.0350 (9)	0.0037 (8)	0.0060 (7)	0.0004 (8)
C2	0.0456 (10)	0.0545 (11)	0.0323 (9)	−0.0030 (9)	0.0102 (8)	−0.0018 (9)
C3	0.0415 (10)	0.0532 (11)	0.0380 (10)	−0.0026 (9)	0.0117 (8)	0.0063 (9)
C4	0.0356 (9)	0.0402 (9)	0.0393 (9)	0.0028 (7)	0.0023 (7)	0.0036 (7)
C5	0.0458 (10)	0.0536 (12)	0.0303 (9)	−0.0018 (9)	0.0045 (8)	0.0007 (8)
C6	0.0417 (10)	0.0518 (11)	0.0341 (9)	−0.0030 (9)	0.0084 (8)	0.0057 (8)
C7	0.0420 (11)	0.0591 (12)	0.0505 (12)	−0.0031 (10)	0.0043 (9)	−0.0030 (10)
C8	0.0425 (11)	0.0548 (12)	0.0533 (12)	−0.0013 (9)	0.0039 (9)	−0.0123 (10)
C9	0.0678 (15)	0.0632 (14)	0.0700 (15)	0.0114 (12)	0.0066 (12)	−0.0006 (12)
C10	0.0706 (16)	0.0697 (15)	0.0857 (19)	0.0242 (13)	0.0016 (14)	−0.0173 (14)
C11	0.0539 (13)	0.0848 (17)	0.0624 (15)	0.0074 (13)	0.0029 (11)	−0.0267 (13)
C12	0.0834 (17)	0.0808 (17)	0.0542 (14)	0.0141 (15)	0.0159 (12)	−0.0067 (13)
C13	0.0661 (14)	0.0672 (14)	0.0548 (13)	0.0171 (12)	0.0086 (11)	−0.0087 (11)
C14	0.0808 (18)	0.127 (3)	0.0847 (19)	0.0258 (18)	0.0152 (15)	−0.0400 (18)
O5	0.1131 (15)	0.0646 (10)	0.0485 (9)	0.0000 (10)	0.0078 (10)	−0.0005 (9)

### (E)-N'-(4-Methylbenzylidene)-4-nitrobenzenesulfonohydrazide monohydrate (III) Geometric parameters (Å, º)

**Table:**

S1—O2	1.4230 (13)	C6—H6	0.9300
S1—O1	1.4284 (13)	C7—C8	1.465 (3)
S1—N1	1.6335 (17)	C7—H7	0.9300
S1—C1	1.7652 (18)	C8—C13	1.379 (3)
O3—N3	1.221 (2)	C8—C9	1.381 (3)
O4—N3	1.220 (2)	C9—C10	1.383 (3)
N1—N2	1.398 (2)	C9—H9	0.9300
N1—H1N	0.850 (15)	C10—C11	1.365 (3)
N2—C7	1.263 (3)	C10—H10	0.9300
N3—C4	1.473 (2)	C11—C12	1.375 (3)
C1—C2	1.383 (2)	C11—C14	1.514 (3)
C1—C6	1.389 (2)	C12—C13	1.372 (3)
C2—C3	1.382 (3)	C12—H12	0.9300
C2—H2	0.9300	C13—H13	0.9300
C3—C4	1.375 (2)	C14—H14A	0.9600
C3—H3	0.9300	C14—H14B	0.9600
C4—C5	1.375 (2)	C14—H14C	0.9600
C5—C6	1.377 (3)	O5—H51	0.808 (16)
C5—H5	0.9300	O5—H52	0.802 (17)
			
O2—S1—O1	120.13 (8)	C1—C6—H6	120.5
O2—S1—N1	107.70 (8)	N2—C7—C8	121.14 (19)
O1—S1—N1	104.45 (8)	N2—C7—H7	119.4
O2—S1—C1	109.10 (8)	C8—C7—H7	119.4
O1—S1—C1	108.56 (8)	C13—C8—C9	117.7 (2)
N1—S1—C1	105.98 (8)	C13—C8—C7	122.4 (2)
N2—N1—S1	113.94 (12)	C9—C8—C7	119.9 (2)
N2—N1—H1N	117.2 (14)	C8—C9—C10	120.4 (2)
S1—N1—H1N	113.8 (15)	C8—C9—H9	119.8
C7—N2—N1	116.04 (17)	C10—C9—H9	119.8
O4—N3—O3	124.16 (17)	C11—C10—C9	121.9 (2)
O4—N3—C4	118.13 (15)	C11—C10—H10	119.0
O3—N3—C4	117.71 (16)	C9—C10—H10	119.0
C2—C1—C6	121.61 (17)	C10—C11—C12	117.2 (2)
C2—C1—S1	119.44 (13)	C10—C11—C14	120.5 (2)
C6—C1—S1	118.77 (13)	C12—C11—C14	122.3 (3)
C3—C2—C1	119.35 (16)	C13—C12—C11	121.9 (2)
C3—C2—H2	120.3	C13—C12—H12	119.1
C1—C2—H2	120.3	C11—C12—H12	119.1
C4—C3—C2	118.22 (16)	C12—C13—C8	120.8 (2)
C4—C3—H3	120.9	C12—C13—H13	119.6
C2—C3—H3	120.9	C8—C13—H13	119.6
C3—C4—C5	123.13 (17)	C11—C14—H14A	109.5
C3—C4—N3	118.79 (15)	C11—C14—H14B	109.5
C5—C4—N3	118.07 (16)	H14A—C14—H14B	109.5
C4—C5—C6	118.66 (16)	C11—C14—H14C	109.5
C4—C5—H5	120.7	H14A—C14—H14C	109.5
C6—C5—H5	120.7	H14B—C14—H14C	109.5
C5—C6—C1	119.01 (16)	H51—O5—H52	113 (3)
C5—C6—H6	120.5		
			
O2—S1—N1—N2	46.42 (15)	C3—C4—C5—C6	−0.8 (3)
O1—S1—N1—N2	175.19 (13)	N3—C4—C5—C6	178.33 (16)
C1—S1—N1—N2	−70.24 (14)	C4—C5—C6—C1	−0.3 (3)
S1—N1—N2—C7	152.32 (14)	C2—C1—C6—C5	0.8 (3)
O2—S1—C1—C2	153.79 (14)	S1—C1—C6—C5	−174.26 (14)
O1—S1—C1—C2	21.22 (17)	N1—N2—C7—C8	175.00 (16)
N1—S1—C1—C2	−90.50 (15)	N2—C7—C8—C13	−4.2 (3)
O2—S1—C1—C6	−31.06 (16)	N2—C7—C8—C9	177.82 (19)
O1—S1—C1—C6	−163.64 (14)	C13—C8—C9—C10	−1.6 (3)
N1—S1—C1—C6	84.65 (15)	C7—C8—C9—C10	176.5 (2)
C6—C1—C2—C3	−0.1 (3)	C8—C9—C10—C11	0.2 (4)
S1—C1—C2—C3	174.89 (14)	C9—C10—C11—C12	1.5 (4)
C1—C2—C3—C4	−1.0 (3)	C9—C10—C11—C14	−180.0 (2)
C2—C3—C4—C5	1.4 (3)	C10—C11—C12—C13	−1.7 (4)
C2—C3—C4—N3	−177.66 (16)	C14—C11—C12—C13	179.8 (2)
O4—N3—C4—C3	163.87 (17)	C11—C12—C13—C8	0.3 (4)
O3—N3—C4—C3	−16.5 (2)	C9—C8—C13—C12	1.4 (3)
O4—N3—C4—C5	−15.3 (2)	C7—C8—C13—C12	−176.6 (2)
O3—N3—C4—C5	164.39 (17)		

### (E)-N'-(4-Methylbenzylidene)-4-nitrobenzenesulfonohydrazide monohydrate (III) Hydrogen-bond geometry (Å, º)

**Table:**

*D*—H···*A*	*D*—H	H···*A*	*D*···*A*	*D*—H···*A*
N1—H1*N*···O5^i^	0.85 (2)	2.00 (2)	2.848 (2)	173 (2)
O5—H51···O2^ii^	0.81 (2)	2.29 (2)	3.006 (2)	148 (3)
O5—H52···O1^iii^	0.80 (2)	2.17 (2)	2.949 (2)	166 (3)
C5—H5···O1^iii^	0.93	2.52	3.167 (2)	127

Symmetry codes: (i) *x*, −*y*+1/2, *z*−1/2; (ii) *x*, *y*+1, *z*; (iii) *x*, −*y*−1/2, *z*+1/2.
